# What Makes It Tick: Exploring the Mechanisms of Post-treatment Lyme Disease Syndrome

**DOI:** 10.7759/cureus.64987

**Published:** 2024-07-20

**Authors:** Kate E Wester, Bianca C Nwokeabia, Rehana Hassan, Taylor Dunphy, Michael Osondu, Carson Wonders, Misbahuddin Khaja

**Affiliations:** 1 School of Medicine, American University of the Caribbean, Cupecoy, SXM; 2 Department of Internal Medicine, BronxCare Health System, Bronx, USA

**Keywords:** autoimmune response, persistent infection, antibiotic treatment, inflammation, post-treatment lyme disease, chronic lyme disease, tick-borne, borrelia burgdorferi, lyme

## Abstract

Post-treatment Lyme disease syndrome (PTLDS), which may also be referred to incorrectly as "chronic Lyme disease," is defined by the Infectious Diseases Society of America (IDSA) as the presence of fatigue, pain, and/or cognitive complaints with the functional impact that persists for more than six months after completing treatment for Lyme disease (LD). These symptoms occur in 10%-20% of patients previously diagnosed with LD caused by the bacteria *Borrelia burgdorferi* and appropriately treated with a course of antibiotics. The symptoms of PTLDS can be easily overlooked or misdiagnosed as a psychiatric manifestation in geographic locations that rarely see LD. In contrast, geographic locations with a higher prevalence of LD may be more aware of PTLDS symptoms and have higher clinical suspicion leading to this diagnosis. The pathophysiology behind the persistent symptoms some people experience from a primary infection is still largely unknown. Some mechanisms that have been proposed include permanent tissue damage and inflammation, immune system dysfunction, autoimmune response, co-infection, and even persistent infection refractory to treatment. We propose that ongoing PTLDS symptoms seem to be related to an autoimmune response to the tissue damage and inflammation caused by the viable or nonviable spirochete pathogen. At this point, PTLDS is diagnosed clinically as no quantifiable methods are available from laboratory or tissue diagnostics as of 2024. Similar pathophysiological features of PTLDS are seen in diseases such as COVID-19 or chronic fatigue syndrome (CFS). More effective diagnostic approaches might include further studies looking at a possible connection in the genomes of individuals developing PTLDS, quantifiable biomarkers, common inflammatory markers/pathways, and careful histopathological studies of human tissues.

## Introduction and background

Lyme disease

Lyme disease (LD) is a complex condition caused by *Borrelia *species found in ticks and mammals [1]. The specific *Borrelia* species varies by location, with *Borrelia burgdorferi *found in North America and *B. afzelii/B. garinii *in Europe and Asia. *Borrelia burgdorferi *relies on hosts, such as ticks and mammals, for essential nutrients [1]. In ticks, *B. burgdorferi *adapts to extreme temperatures and periods of starvation by modulating its protein expression. This modulation includes downregulating outer surface protein A (OspA) to facilitate detachment from the tick midgut and upregulating OspC to enhance its ability to invade the mammalian host during feeding. These changes are essential for the bacterium to endure the harsh conditions within the tick and to transition effectively to a mammalian host, ensuring its survival and ability to establish infection. The disease is transmitted through *Ixodes* tick bites following the habitats and behaviors of the ticks [2]. Once inside the mammalian host, *Borrelia *spp. avoid immune detection and clearance by exploiting components in tick saliva factors that modulate the host immune response [1]. *Borrelia* spp. are mobile spirochete bacilli that use alterations in their surface protein expressions to avoid recognition by the host's immune system. The spirochetes downregulate proteins that are highly immunogenic and upregulate others that help in evading immune responses. Additionally, the bacteria manipulate host proteins to shield themselves from complement-mediated lysis, a part of the innate immune response. They also inhibit antibody-mediated killing by binding to host-derived proteins that prevent the activation of immune pathways that would otherwise lead to their destruction. This ability to manipulate and evade the host's immune mechanisms is crucial for the persistence and dissemination of *Borrelia *spp. within the host, contributing to the pathogenesis of Lyme disease. The clinical manifestations of Lyme disease stem from the inflammatory response rather than from toxins produced by the bacteria.

The incidence of Lyme disease has steadily increased since the 1970s, especially in the United States and Europe [3,4]. The disease's emergence is partially attributed to land-use practices, changing tick habitats and deer populations, and increasing human exposure to infected ticks. The United States sees a concentration of cases in forested regions, particularly in the Northeast and Midwest, with a bimodal age distribution, with peaks among children (ages 5-14) and adults (ages 45-55) [3]. The months of June and July have the highest number of reported cases in the United States [3]. Europe shows similar patterns, with higher incidence rates in forested areas of northeastern and central regions [3]. From 2010 to 2019 in the United States, there were 252,681 confirmed cases [3]. In 2019, it was the sixth most common nationally notifiable disease [3], with one study suggesting that approximately 476,000 cases are diagnosed and treated annually in the United States, and underreporting is common [3].

**Figure 1:**
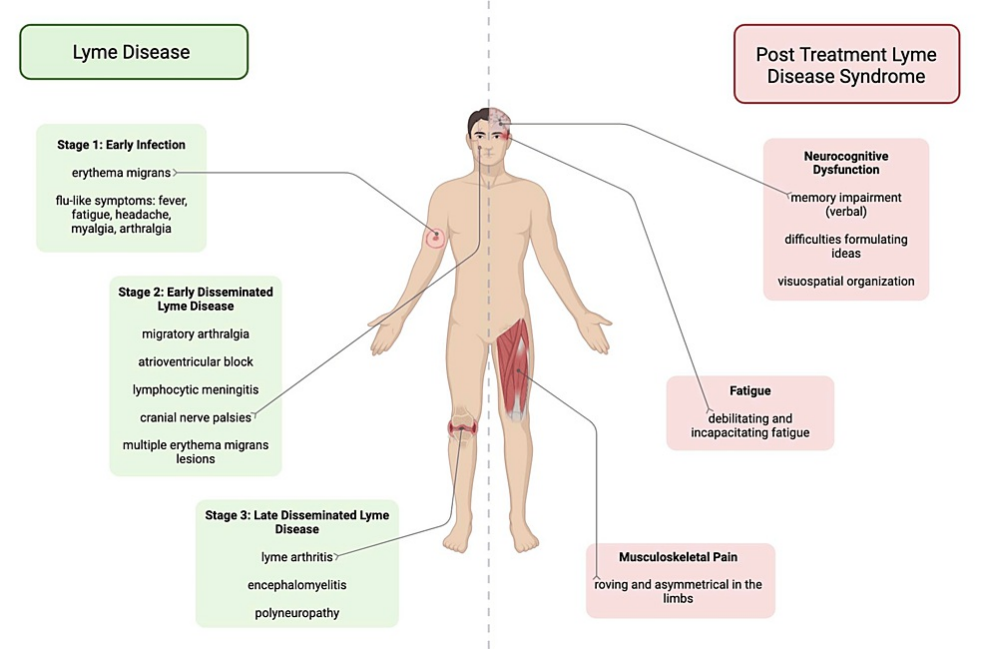
Clinical manifestations of LD versus post-treatment Lyme disease syndrome (PTLDS) Credits: Rehana Hassan

Like syphilis, LD can present as a great imitator with diverse clinical presentations over time [4]. The clinical presentation of classic LD might appear in three phases: early localized, early disseminated, and late Lyme disease (Figure 1). The early localized phase of primary LD is characterized by erythema migrans (EM), a rash that typically appears at the tick bite site within 7-14 days. EM lesions are often found at the site of the tick bite in the axilla or inguinal region and may exhibit a slow expansion over days or weeks, sometimes developing a bull's-eye appearance. The patient might confuse the rash with poison ivy or contact dermatitis until they develop symptoms resembling a viral syndrome, such as fatigue, chills, headache, and muscle and joint pains [2]. On early dissemination weeks to months postinfection, patients might experience multiple EM lesions, painful joint effusions, neurological symptoms such as lymphocytic meningitis, cranial nerve palsies, radiculopathy, or cardiac manifestations such as varying degrees of atrioventricular block. Late Lyme disease can manifest months to years after the initial infection and is characterized by arthritis, particularly in large joints such as the knee, and neurological symptoms such as encephalopathy or polyneuropathy [2,4].

The diagnosis of early localized LD is purely clinically related to the presence of EM, and serological testing is not recommended [2]. Unless the patient was rebitten or experiencing reinfection of Lyme disease, he or she might not develop *Borrelia *antibodies for several days after the appearance of a new, de novo EM Lyme rash [4]. Only if a patient is suspected to have disseminated or late Lyme disease can serological testing be needed to support the diagnosis [2].

Serological testing includes a two-tiered algorithm that "includes an initial enzyme immunoassay (EIA) or immunofluorescence assay (IFA) followed by a Western blot" [2]. Western blots are only performed if the EIA or IFA is positive. An IgM Western blot has three particular bands that can be detected (OspC 24, 39, and 41), and the test is positive if two of the three bands are present [2]. An IgG Western blot has 10 particular bands that can be detected (OspC 18, 23, 28, 30, 39, 41, 45, 58, 66, and 93) and is considered positive if five of the 10 bands are detected [2].

Lyme disease treatment involves using antibiotics such as doxycycline, amoxicillin, or cefuroxime. The treatment duration depends on the disease's stage and its manifestations [5]. Oral antibiotics such as doxycycline, amoxicillin, or cefuroxime are recommended for early localized Lyme disease, with treatment durations ranging from 10 to 14 days. Disseminated Lyme disease, characterized by multiple erythema migrans lesions and neurological and/or cardiac manifestations, may require longer treatment durations, typically 14-21 days or more. In cases of Lyme arthritis, initial treatment involves at least a 28-day course of oral doxycycline, some with a second IV therapy course if necessary [5].

Post-treatment Lyme disease

PTLDS symptoms are broadly defined by prolonged somatic and neurocognitive dysfunction following standard antibiotic treatment for LD (Figure 1). Approximately 10%-20% of LD patients do not respond to standard treatment, and especially with persistent p41 immunoblot seropositivity, they experience the insidious progression to more dermatologic, cardiologic, musculoskeletal, and neurological symptoms [6]. The subjective nature of these symptoms, coupled with the absence of conventional diagnostic biomarkers, makes the identification of PTLDS heavily reliant on a clinical diagnosis. Further treatment becomes undefined, challenging, and primarily symptom-based [7]. Some LD patients can remain serologically positive after completing antibiotic therapy, although the absence of seropositivity does not exclude PTLDS [6,8]. Ultimately, no quantitative biomarker exists to indicate a treatment endpoint and/or definite transition to PTLDS. Risk factors for PTLDS include delayed diagnosis and treatment, incomplete or short-course treatment, and the increased severity of acute LD [6]. Disseminated neurocognitive symptoms such as Bell's palsy, headache, photophobia, other cranial nerve palsies, optic Lyme disease, or neck pain may also increase the risk of persistent symptoms post-treatment. It is reported that having preexisting comorbidities, which may include diabetes, obesity, cardiovascular disease, or chronic pain, during the initial LD infection was predictive of long-term symptoms [8]. 

As mentioned, the initial immune response to infection may also indicate persistent symptoms post-treatment. A muted immune response during acute infection, specifically measured by lower levels of circulating plasmablasts, has been associated with PTLDS. However, this is indicative of a B-cell response and is sensitive but not specific to LD [8,9]. Although there has not been an identifiable pattern in the specific genotypes of the infecting *B. burgdorferi *strain that causes persistent symptoms, it is shown that RST1 strains generate an increased inflammatory response and are associated with greater severity in symptoms and an increased risk of antibiotic-refractory arthritis [8].

Chronic symptoms in PTLDS encompass a constellation of subjective clinical problems: incapacitating fatigue, pain, and neurocognitive dysfunction that persist for more than six months. Symptoms can be intermittent or constant. A previously published definition of PTLDS, which is based on the Infectious Diseases Society of America's (IDSA) proposed case definition, includes the presence of fatigue, pain, and/or cognitive complaints with functional impact calculated by 36-Item Short Form Survey (SF-36) scores, with a composite T score of less than 45 [9,10]. Nevertheless, the symptoms remain subjective. A meta-study published by Cairns and Godwin serves to break down each set of symptoms to establish more definitive diagnostic criteria. The meta-analysis focused on five studies that showed the mean or median time since the diagnosis of LD was three to six years with the persistence of symptoms. When assessing neurocognitive dysfunction, Cairns and Godwin addressed specific tasks such as memory problems, poor concentration, difficulties in formulating ideas, word finding, judgment, and naming objects [11]. Memory problems, particularly verbal memory, were shown to be impaired. Difficulties in formulating ideas were also diminished, which are consistent with results from other studies. Conversely, no increased problems in judgment, conceptual thinking, or naming objects were found. Fallon et al. observed reduced blood flow in discrete white matter areas of the brain in patients with PTLDS compared to healthy subjects. The flow reductions were significantly associated with areas of the brain resulting in deficits in memory and visuospatial organization. The fatigue described in the PTLDS literature is "profound, notable, debilitating, and extreme, not as a vague form of tiredness." The musculoskeletal pain of PTLDS is "roving, asymmetrical pain in the limbs," which should be contrasted to the pain found in a disease such as fibromyalgia where the pain is described as diffuse and symmetrical in many places throughout the body [11].

## Review

Tissue damage and inflammation

Untreated Lyme disease affects multiple tissues, such as nervous tissue, synovial tissue, muscle, and vascular tissue, resulting in tissue damage and inflammation. Tissue damage and inflammation might be primarily due to the host's inflammatory response to the infection rather than the bacterial load at the site of infection, according to Coburn et al. [12]. In PTLDS, the tissue damage and inflammation resulting from infection are thought to persist despite treatment, leading to a variety of chronic symptoms. Inflammation within the nervous system (neuroinflammation) occurs in the central and peripheral nervous systems. Adler et al. claim that either the infection with *B. burgdorferi *activated an immune response that allowed inflammatory mediators to cross the blood-brain barrier and activate CNS immune cells, as well as reactive oxygen species (ROS), causing damage, or the primary inflammatory response directly damaged the blood-brain barrier causing further CNS immune cell activation and continued inflammation [13]. Another study supports the latter mechanism by mentioning changes in miRNA expression in response to *B. burgdorferi *infection [14]. This is significant as miRNAs are involved in cell adhesion, and when upregulated, they can lead to a decrease in tight junctions and a compromised blood-brain barrier.

In addition, within the peripheral nervous system, *B. burgdorferi *infects the cranial nerves, notably the vagus nerve, which serves as an immune regulator. The vagus nerve transmits signals of peripheral inflammation to the CNS, leading to increased cytokines, neuroinflammation, and microglia activation, which has clearly been shown in imaging studies of the CNS for PTLDS. This may also explain why cranial nerve damage from *B. burgdorferi *infection leads to autonomic dysfunction and neuroinflammation seen in PTLDS [13].

As depicted in Lyme arthritis, *B. burgdorferi *also damages the synovial membrane with the persistence of synovial inflammation. A study done with antibiotic-refractory Lyme disease patients found consistent or elevated levels of proinflammatory cytokines in joint and synovial fluid, even though PCR tests for the bacterium DNA were negative in joint fluid [15]. However, negative joint fluid results do not eliminate the possibility that a small portion of *Borrelia *spirochetes has entered the tissues for preservation upon antibiotic treatment [15]. While *B. burgdorferi *spirochetes might be destroyed by antibiotic treatment, its DNA is able to remain in cartilage tissue and bind toll-like receptors, thus contributing to chronic inflammation [16]. Studies have shown that *B. burgdorferi *can cause endothelial damage via the adherence and penetration of endothelial cells in vitro, prompting neutrophil migration and subsequent inflammatory response, but no studies have observed this in humans with PTLDS [13]. However, *B. burgdorferi *spirochetes are known to be mobile, highly invasive, and persistent, which is due in part to their virulence mechanisms and the gene expression that is activated upon infection. *Borrelia *bacteria have developed survival mechanisms that allow them to adhere to the vascular wall, extravasate, and persistently colonize diverse tissue sites [12].

Immune system dysfunction

PTLDS has been postulated to be associated with immune system dysfunction, where the immune response remains dysregulated even after the infection is treated. This theory suggests that the immune system fails to return to normal, leading to ongoing symptoms. A cohort study, as reported by Aucott et al., describes the relevance of chemokine C-C motif ligand 19 (CCL19) during acute infection, which may also pose an immunologic risk factor for PTLDS [17]. This study consisted of a small sample size comparing 76 participants with Lyme disease, and 26 healthy controls followed over 12 months. The results demonstrate that those with a CCL19 level of more than 111.67 pg/mL one month after treatment have a 12-fold higher risk of developing PTLDS within 6-12 months (95% confidence interval). As mentioned previously, *B. burgdorferi *infects the vagus nerve, which regulates the immune system and has an anti-inflammatory effect via its efferent cholinergic signaling. The involvement of this nerve during acute infection may cause nerve damage that contributes to chronic dysautonomia with neuroinflammation [13].

However, another article describes decreased inflammatory response and immune cell trafficking pathways in Lyme disease patients six months after antibiotic treatment [18]. This study analyzed the CSF profile and transcriptome of patients diagnosed with Lyme disease at the time of diagnosis and again six months after antibiotic treatment. Before treatment, there was a change in approximately 1,000 genes compared to a noninfected transcriptome. In the post-treatment analysis, nearly 700 genes were continued to be altered, with approximately 50% of the genes being upregulated. This decrease in inflammatory response may partially be explained through *B. burgdorferi*'s* *effect on the innate and adaptive immune system. The outer surface proteins (OspC, OspA, etc.) on *B. burgdorferi *allow it to inhibit the complement system via the classical, alternative, and lectin pathways [12]. Thus, crippling the immune system as the membrane attack complex, phagocytosis, and the formation of specific antibodies targeted to infection are impaired in the process. As evidenced by the study done on mutant OspC *B. burgdorferi *mice versus wild type, there was a reduced survival of the mutant OspC *B. burgdorferi *strain mice after 30 minutes postinoculation [12].

Autoimmune response

Several studies have shown that Lyme disease and PTLDS have been linked to autoimmune conditions. Arvikar et al. discuss a large cohort of patients experiencing post-Lyme autoimmune joint diseases [19]. Their study identified 30 patients with a median age of 55 years who developed systemic autoimmune joint diseases such as rheumatoid arthritis (RA), psoriatic arthritis (PsA), and peripheral spondyloarthritis (SpA) within four months of their LD diagnosis [19]. Of these patients, 27 of them had adequate sera to test for LD-associated autoantibodies such as endothelial cell growth factor (ECGF), apolipoprotein B-100 (ApoB-100), and matrix metalloproteinase-10 (MMP-10). Ten patients tested positive for at least one of the autoantibodies, and it was also noted that the levels of anti-ECGF antibodies in the sera correlated with obliterative microvascular lesions in synovial tissue indicating pathologic potential. This autoantibody positivity was significantly greater in patients with a history of LD than in healthy controls or patients with a history of RA/PsA/SpA with no history of LD. Additionally, the article by Yehudina and Trypilka reports a case where systemic lupus erythematosus (SLE) developed in a patient several months after they completed treatment for Lyme disease, alluding to a potential link between LD and autoimmune disorders [20].

In a related study, Chandra et al. [21] measured the levels of antibodies against neural proteins in PTLDS patients with the intent of finding an immune abnormality that explained the neurological and cognitive symptoms described in PTLDS. These levels were compared to healthy controls and patients who had successfully recovered from LD. This demonstrated a notable disparity in the levels of anti-neural antibodies among patients afflicted with PTLDS as they exhibited substantially elevated levels (49%) compared to the two other cohorts. Firstly, healthy individuals showed a significantly lower prevalence (15%) of these antibodies. Secondly, individuals who had previously contracted Lyme disease but successfully recovered displayed an intermediate prevalence (18.5%) of anti-neural antibodies. This finding underscores a potential autoimmune response that may have a role in the pathogenesis of neurological symptoms experienced in PTLDS. The study did not determine a definitive mechanism by which neural antibodies are created. However, it considered two speculations as most likely. The first mechanism proposed is the release of autoantigens from neural tissue damage during active infection. This process causes posttranslational protein modification, creating new self-epitopes that attack the nervous system. The second mechanism involves the potent mitogenic effect of *Borrelia* antigens, such as OspA and OspB, on polyclonal B cells. This effect leads to an increase in antibody-secreting cells. 

Persistent infection

Another suggested mechanism for PTLDS is the persistence of infection by *B. burgdorferi*. In vitro research demonstrates how *B. burgdorferi *can form persister organisms that are less likely to be eradicated by standard antibiotics. These organisms form either under antibiotic pressure or during stationary phase growth; thus, antibiotic exposure drives persistence in the host. In mouse, dog, and non-human primate models, *B. burgdorferi *persistence is measured by tissue histopathology and PCR in animals treated with antibiotics [8]. The mouse model is deemed the most useful system for studying *B. burgdorferi *infection in mammals due to their genomic similarity to humans, ability to mimic aspects of disease pathology, ease of handling, and ethical considerations. In mouse studies, disease severity was impacted by the strain of the mouse, which points to a possible genetic susceptibility requirement in the host [22]. Using the same mouse model and xenodiagnoses with ticks, researchers showed that spirochetes were detectable for up to three months after treatment with either ceftriaxone or doxycycline for *B. burgdorferi*. The spirochetes recovered from antibiotic-treated mice lacked genes on plasmids 1p25 and 1p28-1, which are associated with infectivity. This points to the possible attenuation of genes in the surviving spirochetes [22]. A subsequent study demonstrated that rare spirochete-like forms could be detected by immunohistochemistry in connective tissue within the heart and tibiotarsal joints of antibiotic-treated mice, although the spirochetes could not be directly cultured [23,24]. The findings of these consecutive studies suggest that attenuated but non-cultivable *B. burgdorferi *spirochetes can persist at low levels after antibiotic treatment in mice. It is possible that similarly attenuated but non-cultivable *B. burgdorferi *spirochetes may also persist in human cartilage or other deposits, attributing to the symptoms of PTLDS. This study also postulated that some *B. burgdorferi *DNA could remain intact if it is sequestered in cellular debris such as green fluorescent protein (GFP) deposits such as cartilage [23].

Bockenstedt et al. suggest a possible mechanism in that *B. burgdorferi *persists in human hosts by transforming into cysts. In vitro studies show that *B. burgdorferi *can alter its morphology under nutrient deprivation [22]. However, the formation of true bacterial cysts and endospores requires structural changes that occur over hours to days, not just the rapid times found in vitro. Although this supports persistent infection, levels at six months or longer were not tested in these studies, which is the minimum time frame stated by the IDSA's case definition for persistent symptoms of PTLDS.

Few studies have tested further than four months or within chronic disease; however, a study by Hodzic et al. aimed to determine if mice could be positively tested for spirochetes following the later administration of antibiotics and during the chronic stage of infection [23]. While this is still not considered a sufficient timeline for PTLDS, this study was the first of its kind to focus on a longer period and specifically aimed at extending the findings of the previous study by Bockenstedt et al. [22]. The data gathered by Hodzic et al. supported the conclusion that antibiotic treatment resulted in the persistence of low numbers of spirochetes in tissues of treated mice and that ticks could acquire and transmit very low levels of infectious spirochetes, thus indicating that these persisting spirochetes retain their infectivity to some extent. The study also concluded that quantitative PCR (qtPCR) results demonstrated very low copy numbers of spirochetal DNA, in contrast to the "spirochetal burst" phenomenon of replication normally seen after tick feeding and initial infectivity. This supports the idea that spirochetes can be infectious but have an altered ability to replicate due to a possible change in the genome or due to some susceptibility in the host [23]. The remaining spirochetes that have the ability to infect but not replicate may also release lipoproteins, which is both an acute and chronic process. Spirochetal lipoproteins may induce a prolonged proinflammatory response, which can present as the constitutional symptoms seen in PTLDS [24]. They then extensively searched for intact, antigen-expressing spirochetes in specific tissues via immunohistochemistry. They found that collagen is an ideal niche for spirochete survival and possibly immune evasion. In addition to ligaments and tendons of the tibiotarsal region and other joint tissues, there are vessels at the base of the heart that are collagen-rich preferential regions for spirochetes in the persistent phase of infection. Hodzic et al. clarified that their studies were performed on mice, and conclusions were based on DNA amplification [23].

Therefore, more data needs to be gathered on antibiotic-treated human patients. In an effort to bridge this gap, Hodzic et al. [23] compared their results to a handful of small studies in antibiotic-treated human patients that investigated the persistence of *B. burgdorferi *in collagenous tissue, including ligamentous, synovial, and skin tissues. The first study documented the persistence of *B. burgdorferi *in tendon tissue, specifically the flexor retinaculum, causing musculoskeletal deformities and tendinous pain in the single subject that was studied. They identified spirochetes situated between collagen fibers and along fibroblasts of the ligamentous tissue in this subject. However, this study was limited due to the single subject used and the lack of follow-up [24]. The second study contained four human subjects with treatment-resistant Lyme arthritis after antibiotic therapy. It sought to identify the intra-articular presence of *B. burgdorferi *in synovial fluid and synovial membrane. They concluded that even if the patients had negative PCR synovial fluid results, synovial membrane tissue should be analyzed as a source of persistent infection, as they were able to obtain positive synovial membrane tissue PCR samples even when the synovial fluid was negative, and the patients had persistent symptoms [25]. Future studies in humans must examine not only synovial and CSF fluids but also bone, joint, and nerve tissues. This may be achieved through postmortem analyses or by studying tissue removed during hip and knee replacements.

Co-infections

In addition to immune dysfunction, autoimmune response, and persistent infection affecting PTLDS, tick-borne co-infections might contribute to the pathophysiology of PTLDS. These co-infections, often accompanying LD, exacerbate disease expression and impede therapeutic success. A case series by Trouillas and Franck highlights 10 patients with severe neurological limb paralysis in patients with mixed infections of *Borrelia*, *Babesia divergens*, *Anaplasma phagocytophilum*, and *Bartonella *[26]. Another study that conducted long-term treatment combining antibiotics and anti-parasitics resulted in complete motor recovery in seven out of 10 cases, suggesting the importance of alternative management for co-infections [13]. This study supports the notion that a different antibiotic regimen may be necessary to address persistent infections and co-infections, as proposed by Adler et al. shedding light on potential reasons for post-treatment Lyme disease persistence [13].

In a study, Berghoff suggested that diagnostic challenges arise due to overlapping symptomatology between Lyme borreliosis and co-infections, necessitating nuanced analysis for comprehensive identification [27]. However, diagnostic and therapeutic options for chronic infectious diseases, including co-infections, remain limited. The intracellular localization of pathogens, excluding *Borrelia burgdorferi*, requires antibiotics with intracellular activity, yet treatment failure rates remain high. Both tick-borne and non-tick-borne co-infections, including bartonellosis, tularemia, and *Mycoplasma* infections, pose diagnostic and therapeutic challenges [27]. The notable co-infections associated with Lyme disease arise from a range of pathogens, predominantly including *Bartonella henselae *among various *Bartonella *species, as well as *Chlamydia trachomatis*, *Chlamydophila pneumoniae*, *Yersinia enterocolitica*, and *Mycoplasma pneumoniae*. Berghoff conducted several laboratory diagnostic tests for indirect pathogen detection, including serological tests and lymphocyte transformation tests (LTT, synonym lymphocyte proliferation test {LPT}) [27]. Previous infection can be confirmed with serological tests, but a positive serological finding does not prove that the infection caused the current illness. Thus, the presence of active infection cannot be determined, but it also cannot be excluded in the case of seronegativity. Only if positive laboratory findings or deterioration occurs in a temporal relationship with the disease state and development can the assumption of chronic disease be justified, e.g., in cases with previous seronegativity, negative LTT, or significantly lower initial values. The frequency of seropositivity and positive LTT of co-infections was evaluated. Interestingly, *Bartonella henselae *showed the highest positive serology level at 78%, but its percentage for LTT was not completed. In contrast, *Chlamydia trachomatis*'s positive serology level was 5%, but its percentage for LTT was 100% [27].

Discussion

Understanding the etiology of PTLDS presents a challenge due to the need for more reliable evidence to elucidate the mechanism of the condition. Several possible etiologies have been suggested, but none have had conclusive support. PTLDS might be due to an autoimmune response to tissue damage and/or direct inflammation caused by *Borrelia *spirochetes or bacterial fragments. Immune dysfunction caused by *B. burgdorferi *at the innate and adaptive level may weaken the immune system, thereby promoting persistent infection. The lipoprotein antigens associated with Lyme disease are highly inflammatory, and the retained antigens have been seen in mouse models. This may lead to the characteristic symptoms of severe fatigue, musculoskeletal pain, and cognitive symptoms [17].

Several diseases exhibit similar pathophysiological features to post-treatment Lyme disease syndrome, offering potential insights into its mechanisms. Myalgic encephalomyelitis/chronic fatigue syndrome (ME/CFS) shares PTLDS's hallmark symptoms of debilitating fatigue, cognitive difficulties, and sleep disturbances [28]. However, unlike PTLDS, ME/CFS often lacks a clear infectious trigger and presents with additional symptoms such as post-exertional malaise [29]. Similarly, post-viral fatigue syndrome (PVFS) can follow viral infections, such as COVID-19, causing persistent fatigue and cognitive impairment that mirror PTLDS. However, PVFS typically has a shorter course compared to PTLDS, resolving within six months in most cases [30]. Finally, Epstein-Barr virus (EBV) infection, particularly chronic active Epstein-Barr virus (CAEBV), can present with overlapping symptoms such as fatigue, pain, and cognitive issues [31]. However, CAEBV is often distinguished by persistent viral reactivation detectable through EBV-DNA or specific antibodies, along with characteristic clinical features that are not typically observed in PTLDS [32].

There are limited in vivo studies in humans analyzing persistent infection. Although non-human mammalian studies have demonstrated that there may be a possible genetic susceptibility requirement in the host, this was only performed on mouse models and related to disease severity, which is a risk factor for developing PTLDS. Disease severity differs greatly between mice and humans when it comes to measuring and subsequently reporting somatic and neurocognitive symptoms. The limited case reports in humans sought to identify spirochetes in specific tissues and pointed toward collagen being an ideal survival medium. While this finding is a step in the right direction, additional studies with larger amounts of subjects are required to increase the power of these studies.

## Conclusions

This literature review seeks to shed light on the multifaceted nature of post-treatment Lyme disease syndrome, giving possible insights into its elusive pathophysiology. Despite considerable advancements in understanding Lyme disease etiology, PTLDS remains a complex entity with significant implications for affected individuals' quality of life. This review emphasizes the importance of recognizing PTLDS as a distinct clinical entity, which is characterized by persistent somatic and neurocognitive dysfunction following conventional treatment for Lyme disease. Mechanisms possibly implicated in PTLDS include tissue damage and inflammation, immune system dysfunction, autoimmune response, persistent infection by *B. burgdorferi*, and the complications of co-infection with other microorganisms. Moreover, this review identifies key challenges in the diagnosis and management of PTLDS, including the lack of specific biomarkers, clinical features shared with other conditions, and limited therapeutic options. Future research endeavors should focus on elucidating the interplay between host factors, bacterial persistence, and immune dysregulation to pave the way for more effective diagnostic strategies and personalized treatment approaches.
